# Balancing the Risk-Benefit Ratio of Immune Checkpoint Inhibitor and Anti-VEGF Combination Therapy in Renal Cell Carcinoma: A Systematic Review and Meta-Analysis

**DOI:** 10.3389/fonc.2021.739263

**Published:** 2021-10-14

**Authors:** Li Tao, Huiyun Zhang, Guangyu An, Haoning Lan, Yaoqi Xu, Yang Ge, Jiannan Yao

**Affiliations:** ^1^ Beijing Chaoyang Hospital, Capital Medical University, Beijing, China; ^2^ Department of Oncology, Beijing Chaoyang Hospital, Capital Medical University, Beijing, China

**Keywords:** combination therapy, renal cell carcinoma (RCC), immune checkpoint inhibitors (ICI), efficacy, safety, VEGF targeted therapy

## Abstract

**Background:**

Although immune checkpoint inhibitors (ICIs) combined with vascular endothelial growth factor receptor (VEGFR)-targeted therapy and sunitinib monotherapy have been widely applied to metastatic renal cell carcinoma (mRCC), effectiveness and safety data are still lacking. To optimize clinical decision-making, we conducted a systematic review and meta-analysis of published randomized clinical trials to characterize the efficacy and the risk of adverse events (AEs) in patients treated with ICIs plus anti-VEGF therapy.

**Materials and Methods:**

We used PubMed, EMBASE, and the Cochrane Library to retrieve randomized controlled trials (RCTs) published before March 27, 2021. The efficacy outcomes were progression-free survival (PFS), overall survival (OS), and objective response rate (ORR). The pooled risk ratio (RR) and 95% confidence intervals (CI) of AEs were calculated in the safety analysis.

**Results:**

Six RCTs involving 4,227 patients were identified after a systematic search. For OS, ICI and anti-VEGF combination therapy decreased mortality approximately 30% in the intention-to-treat population (ITT) (hazard ratio (HR) = 0.70, 95% CI: 0.57–0.87), but there was no statistical difference in patients evaluated as “favorable” by the International Metastatic Renal-Cell Carcinoma Database Consortium (IMDC) criteria compared with monotherapy (HR = 0.90, 95% CI: 0.55–1.46, *p* = 0.66). In terms of PFS, the progression risk for all participants declined 35% (HR = 0.65, 95% CI: 0.50–0.83) and patients evaluated as “poor” by IMDC benefited further (HR = 0.46, 95% CI: 0.36–0.58). No evident divergence was found in age and sex subgroups. The RRs of all-grade hypertension, arthralgia, rash, proteinuria, high-grade (grades 3–5) arthralgia, and proteinuria developed after combination therapy were increased compared with sunitinib. The risk of high-grade hypertension and rash showed no statistical difference. However, the risk of hand-foot skin reaction (HFSR), stomatitis, and dysgeusia decreased in combination therapy groups.

**Conclusions:**

Compared with sunitinib, OS, PFS, and ORR were significantly improved in patients receiving ICI and anti-VEGF combination therapy at the expense of increased specific AEs. More attention should be paid to individualized application of these combination therapies to achieve the best benefit-risk ratio in the clinic.

**Systematic Review Registration:**

[https://inplasy.com/] INPLASY: 202130104.

## 1 Introduction

Renal cell carcinoma (RCC) is the most common renal neoplasm (1) and approximately 30% of patients present with metastatic disease (2), thus, aggravating the mortality of RCC. In the last decade, medical treatment for RCC has laid great emphasis on vascular endothelial growth factor receptor (VEGFR) monoclonal antibody and tyrosine kinase inhibitors (TKI) (3). These VEGF targeted therapies have improved clinical outcomes by suppressing endothelial cell proliferation and reforming carcinoma vasculature. Depending on treatment type, metastatic RCC response rates can reach 30%, and median overall survival can reach up to 2 years (4). Extensive clinical research has shown that sunitinib, a widely used VEGF inhibitor, is associated with drug resistance and numerous adverse events which may lead to frequent treatment withdrawal (4–6). Additionally, 63% of patients receiving sunitinib reported grade 3 or higher adverse events (AEs) including hypertension, rash, fatigue, and hand-foot skin syndrome (HFSR) (7).

Recently, the development and approval of immune checkpoint inhibitors (ICIs) has altered the treatment paradigm for RCC (8). Agents that target cytotoxic T lymphocyte-associated molecule-4 (CTLA-4), programmed cell death receptor-1 (PD-1), and programmed cell death ligand-1 (PD-L1) are the most widely studied and recognized (9). However, these agents are broadly associated with ill-defined AEs, referred to as immune-related adverse events (irAEs), and characterized by clinical manifestations similar to autoimmunity disorders (10). Moreover, long-term exposure to ICIs can cause primary or secondary resistance (11). The leading underlying mechanisms for resistance include neoantigen loss, defect of antigen presentation, alternative immune checkpoints, and defective interferon signaling.

In order to address these concerns, multiple research groups are actively seeking effective treatments for RCC, as evidenced by 321 clinical trials listed at ClinicalTrials.gov as of February 10, 2020, including combination therapy of anti-VEGF and ICIs (8). Antiangiogenics (such as cabozantinib and axitinib) with pleiotropic immunomodulating properties, combined with immunotherapies, are preferred to traditional monotherapy (4). In RCC, the von Hippel-Lindau (VHL) gene is often silenced or lost, which drives the development of a highly vascularized pathology. Notably, PD-1 and its ligands are reported to be expressed on kidney macrophages, dendritic cells, lymphocytes, and renal proximal tubule epithelial cells (12). These two factors contribute to the immune-suppressive microenvironment. Thus, the combination of ICIs and VEGF targeted therapy offers synergistic improvements (7). However, the optimal combination regimen and sequence of treatments will likely continue to evolve as novel therapeutic agents and combinations gain FDA approval (13). To date, both combination therapy with ICIs and anti-VEGF or sunitinib monotherapy have been recommended in the revised National Comprehensive Cancer Network (NCCN) guidelines.

Despite the demonstrated success of combination therapy, several important questions remain unresolved. Will the combination of ICIs and anti-VEGF improve the prognosis at a cost of increased toxicity? Are there any clinical factors that could guide decision making in order to prolong effective treatment and maintain patient quality of life (QoL)? Based on the remarkable efficacy shown previously and the recent findings of the randomized controlled trials with combination therapy, we performed a systematic review and meta-analysis to further evaluate the impact of ICIs and anti-VEGF combination therapy on the clinical outcomes of RCC patients. Our findings catalog the frequency and severity of the most common AEs, including hypertension, arthralgia, rash, proteinuria, HFSR, stomatitis, and dysgeusia, which might lead to treatment withdrawal and severe clinical consequences (14–22).

## 2 Methods

### 2.1 Search Strategy

We performed a systematic search for associated studies published before March 27, 2021, in Pubmed, Embase, and the Cochrane Library. The search terms were as follows: “renal carcinoma/exp” and “randomized controlled trial/exp” and (“vasculartropin/exp” or “anti-angiogenesis/exp” or “angiogenesis inhibitor/exp”) and (“immune checkpoint inhibitor/exp” or “programmed cell death protein 1/exp” or “programmed cell death ligand protein 1/exp” or “cytotoxic T-lymphocyte-associated protein 4/exp”) and “human/exp”. No language limitation was applied, and all adopted studies were screened manually from the reference list and other relevant articles. Two reviewers (LT and HZ) independently searched and assessed the content and quality. Any disagreement was resolved by the corresponding author. The PRISMA statement is displayed in **Supplementary Table 1**.

### 2.2 Study Selection

The inclusion criteria were as follows: (1) Patients who were diagnosed with RCC or had untreated advanced RCC with a clear-cell component and at least one measurable lesion according to the Response Evaluation Criteria in Solid Tumors (RECIST); (2) Karnofsky performance status score of at least 70 (scores range from 0 to 100, with lower scores indicating greater disability); (3) Adults (18 years old or older); (4) adequately controlled blood pressure, with or without medications; and adequate organ function; (5) patients without previous systemic therapy for advanced disease; (6) studies reported with efficacy, including overall survival (OS), progression-free survival (PFS), objective response rate (ORR), and associated AEs; (7) randomized controlled trial studies; and (8) when results from an RCT were reported and analyzed more than once, the primary data were included.

The exclusion criteria were as follows: (1) not related to RCC; (2) reviews, meta-analysis, case reports, letters, or expert opinions; (3) single arm; (4) insufficient data; (5) experimental group did not receive combination therapy of ICIs and anti-VEGF; (6) duplicates; (7) studies that enrolled patients younger than 18 years old or animals; and (8) not RCTs.

### 2.3 Data Extraction and Risk of Bias Assessment

Two reviewers (LT and HZ) independently extracted the data, and any disagreement was settled through discussion. The following data from eligible studies were collected: National Clinical Trial (NCT) number, first author, treatment arms, control arms, the overall number of patients, publication year, enrollment criteria, characteristics of patients, outcomes, study methods, and number of selected adverse events. The risk of bias was assessed by the Cochrane Collaboration and was classified as “low”, “unclear”, or “high” in several areas.

### 2.4 Outcome Measures

Outcomes for efficacy were evaluated by PFS, OS, and ORR (defined by the Response Evaluation Criteria in Solid Tumors), and safety was evaluated by events of selected AEs. The severity of AEs was graded according to the National Cancer Institute Common Terminology Criteria for Adverse Events, version 4.0.

### 2.5 Statistical Analysis

The hazard ratios (HR) were represented with 95% confidence intervals (CI) for generic inverse variance outcomes, and risk ratios (RR) were shown with 95% confidence intervals for outcomes. We adopted mean values for continuous outcomes.

Statistical heterogeneity across trials or subgroups was tested using the *I* ^2^ testing. As six of the trials were multicenter, the random effects model was adopted in all analyses to balance the effect of each study, and all included studies were equally weighted (23). The Inverse-Variance (I-V) pooling model was applied to analyze OS, PFS, and ORR, while the Mantel-Haenszel (M-H) pooling model was adopted in the analysis of adverse events. An *I* ^2^ >50% implied significant heterogeneity (24). Subgroup analysis and sensitivity analysis were performed where appropriate. Subgroup analysis was conducted for the primary outcomes: (1) subgroups with different evaluations from the IMDC; (2) PD-L1-positive or PD-L1-negative subgroups; (3) age subgroup (divided by the age of 65); and (4) sex subgroup.

## 3 Results

**Figure 1:**
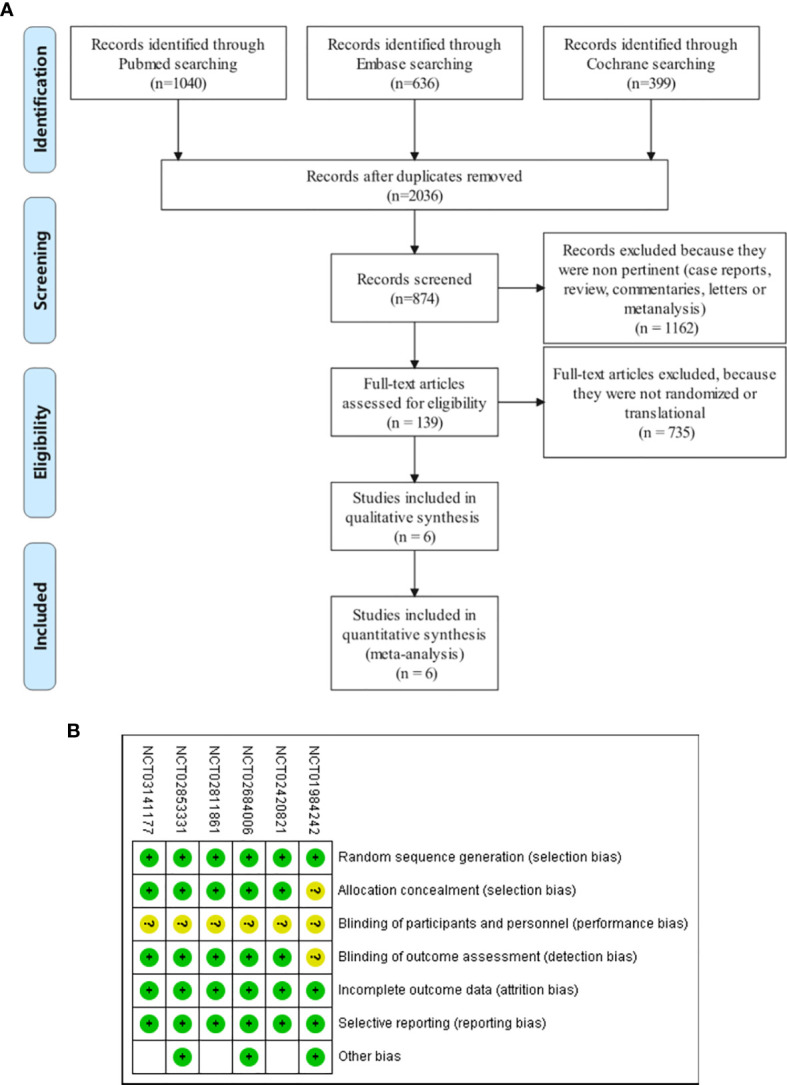
**(A)** Flow diagram of study selection. Database searching was based on PubMed, EMBASE, and the Cochrane Library. **(B)** Quality assessment for six included studies. Quality of trials was categorized into three grades: low risk of bias (+), high risk of bias (–), and unclear (?).

A total of 3,042 studies were identified, of which 1,006 were duplicates. We scanned titles and abstracts and excluded 1,897 articles for not meeting the inclusion criteria. Having obtained full-text articles for 139 citations, we excluded 133 for non-RCT. Finally, six articles involving 4,227 participants were adopted in this systematic review and meta-analysis. The selection flow diagram is shown in **Figure 1A**.

### 3.1 Study Characteristics

The final analysis included six RCTs published between 2018 and 2021, all with sunitinib as the control arm. All the patients in these trials had never received any systematic anticancer therapy for RCC. Atezolizumab plus bevacizumab was applied as the treatment arms in NCT01984242 and NCT02420821. In other RCTs, different treatment combinations were adopted. Six trials researched the influence of PD-L1 expression on PFS. Five trials explored the impact of PD-L1 expression on OS. Overall, 4,227 participants were available for PFS and ORR and 4,025 for OS. Characteristics of included studies are shown in **Table 1**.

**Table 1:** Characteristics of the included studies.

NCT	NCT01984242 (25)	NCT02420821 (26)	NCT02684006 (27)	NCT02811861 (28)	NCT02853331 (29)	NCT03141177 (30)
Study	Immotion150	Immotion151	Javelin Renal 101	—	Keynote-426	CheckMate 9ER
Year	2018	2019	2020	2021	2019	2021
Author	McDermott, D. F.	Rini, B. I.	Motzer, R. J.	Motzer, R. J.	Rini, B. I.	Choueiri, T. K.
Treatment arms	Atezolizumab+ Bevacizumab*	Atezolizumab+ Bevacizumab	Avelumab+ Axitinib	Pembrolizumab+ Levatinib**	Pembrolizumab+ Axitinib	Nivolumab+ Cabozantinib***
Control	Sunitinib	Sunitinib	Sunitinib	Sunitinib	Sunitinib	Sunitinib
Number of patients	101 vs. 101	454 vs. 461	442 vs. 444	355 vs. 357	432 vs. 429	323 vs. 328
Median age(years)	62 vs. 61	62 vs. 60	62 vs. 61	64 vs. 62	62 vs. 61	62 vs. 61
Sex (male% / female%)	73/27 vs. 78/22	70/30 vs. 76/24	71/29 vs. 77/23	72/28 vs. 77/23	71/29 vs. 75/25	77/23 vs. 71/29
PD-L1 +(% of patients)	50 vs. 59	49 vs. 40	55 vs. 25	30 vs. 33	59 vs. 62	26 vs. 25
Prognostic model	MSKCC	MSKCC	IMDC	IMDC**** and MSKCC	IMDC	IMDC
Favorable risk %	30 vs. 21	20 vs. 20	21 vs. 22	31 vs. 35	32 vs. 30	23 vs. 22
Intermediate risk %	61 vs. 69	69 vs. 69	61 vs. 62	59 vs. 54	55 vs. 57	58 vs. 57
Poor risk %	9 vs. 10	11 vs. 11	16 vs. 16	9 vs. 10	13 vs. 12	19 vs. 21
Primary endpoints	PFS	OS, PFS	OS, PFS	OS, PFS	OS, PFS	OS, PFS
Median PFS (months)	11.7 vs. 8.4	11.2 vs. 8.4	13.8 vs. 8.4	23.9 vs. 9.2	15.1 vs. 11.1	16.6 vs. 8.3
Median OS (months)	NR	33.6 vs. 34.9	NR	NR	NR	NR
ORR	NR	151/454 vs. 144/460	227/442 vs. 114/444	252/355 vs. 129/357	256/432 vs. 153/429	180/323 vs. 89/328

NR not reported, PFS progression-free survival, OS overall survival, ORR objective response ratio.

* Atezolizumab alone arm was not considered.

**Levatinib and Everolimus combination arm was not considered.

***Nivolumab, Ipilimumab and Cabozantinib combination arm was not considered.

****Only IMDC was adopted in our analysis

### 3.2 Risk and Bias

All six trials had an unclear risk of performance bias because their design was open label **(** **Figure 1B** **)**. Due to the absence of allocation design and independent assessment institution results in one trial (NCT01984242), the selection and detection bias were determined to be unclear. Publication bias was evaluated by constructing a funnel plot in the meta-analysis of the all-grade adverse events. Begg’s test standardizes the effect size by subtracting the weighted mean and dividing it by the standard error, and then verifies whether the effect size is correlated with the standard error by correcting the rank correlation analysis (31). Begg’s test was assessed by funnel plot asymmetry, and *p* < 0.05 was defined as significant publication bias **(** **Supplementary Figure 1** **)**. Egger’s regression test uses linear regression to measure the symmetry of inverted funnel plot according to the natural log of ratio, and the intercept of the line represents the degree of asymmetry (32). If *p* > 0.05, there is no publication offset **(** **Supplementary Figure 2** **)**. Only the *p*-value of Egger’s test for HFSR (*p*>|*t*| = 0.045) showed obvious publication bias, likely owing to the inadequate included articles (*n* < 10). Review Manager Version 5.2 (Cochrane IMS, Oxford, UK) and Stata/SE 16.0 was used to conduct statistical analysis.

### 3.3 Efficacy

#### 3.3.1 Overall Survival

**Figure 2:**
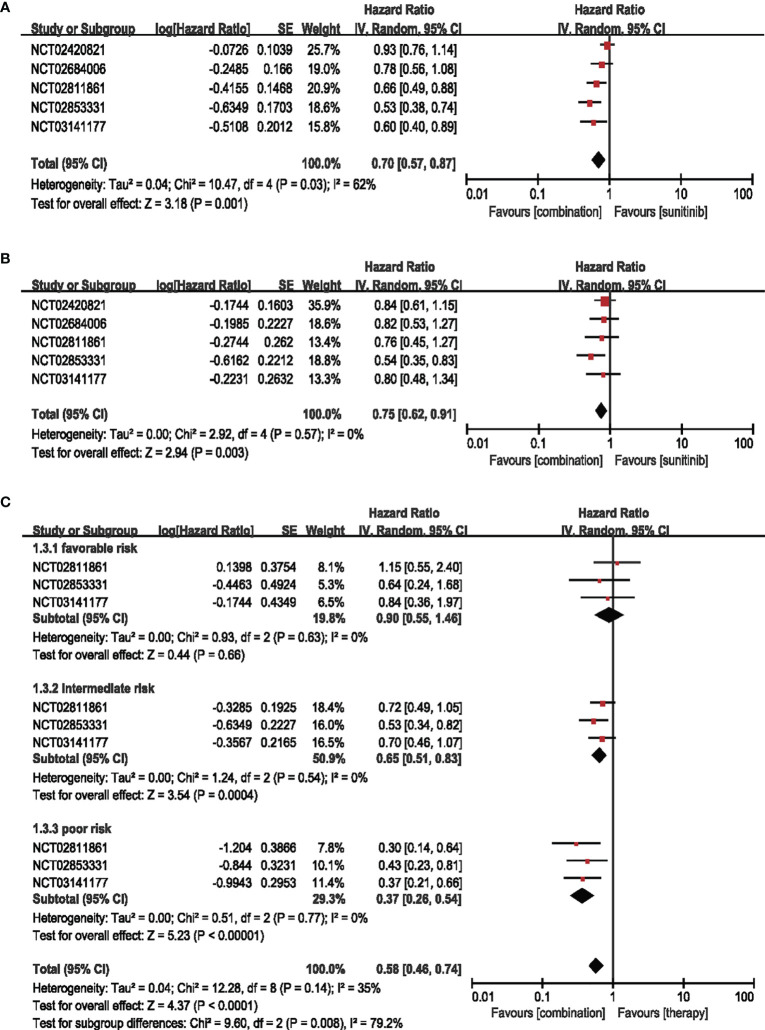
**(A)** Forest plot of OS in patients treated with combination therapy of ICIs and anti-VEGF *vs.* sunitinib monotherapy. **(B)** Forest plot of PD-L1-positive patients treated with combination therapy of ICIs and anti-VEGF *vs.* sunitinib monotherapy. **(C)** Forest plot of different IMDC-evaluated patients treated with combination therapy of ICI and anti-VEGF *vs.* sunitinib monotherapy.

Five studies that included 2006 participants from the combination group and 2019 participants from the sunitinib groups examined the overall survival by HR. Combination therapy of ICIs and anti-VEGF decreased the risk of death relative to sunitinib alone by 30% (HR = 0.70, 95% CI: 0.57–0.87, *p* = 0.001; *I* ^2^ = 62%) **(** **Figure 2A** **)**. We performed the subgroup analysis in four dimensions to further investigate the potential factors contributing to the outcomes.

##### 3.3.1.1 OS in Patients With PD-L1-Positive Expression (≥1%)

Five articles were adopted to analyze the OS in the patients with PD-L1-positive expression (≥1%). The risk of death in combination therapy was decreased by 25% compared with sunitinib monotherapy (HR = 0.75, 95% CI: 0.62–0.91, *p* = 0.003; *I* ^2^ = 0%) **(** **Figure 2B** **)**.

##### 3.3.1.2 Subgroup Analysis OS by Age, Sex, and IMDC

Three trials were enrolled for IMDC evaluation concerning age and sex subgroups. No significant difference was detected in the age and sex subgroups **(** **Supplementary Figure S3** **)**. For IMDC evaluation, the combination therapy showed little contribution (HR = 0.90, 95% CI: 0.55–1.46, *p* = 0.66; *I* ^2^ = 0%) in the favorable group, while showing decreased risk of death by 35% in the intermediate-risk subgroups (HR = 0.65, 95% CI: 0.51–0.83, *p* = 0.0004; *I* ^2^ = 0%) and 63% in the poor-risk subgroups (HR = 0.37, 95% CI: 0.26–0.54, *p* < 0.00001; *I* ^2^ = 0%) **(** **Figure 2C** **).**

#### 3.3.2 PFS

**Figure 3:**
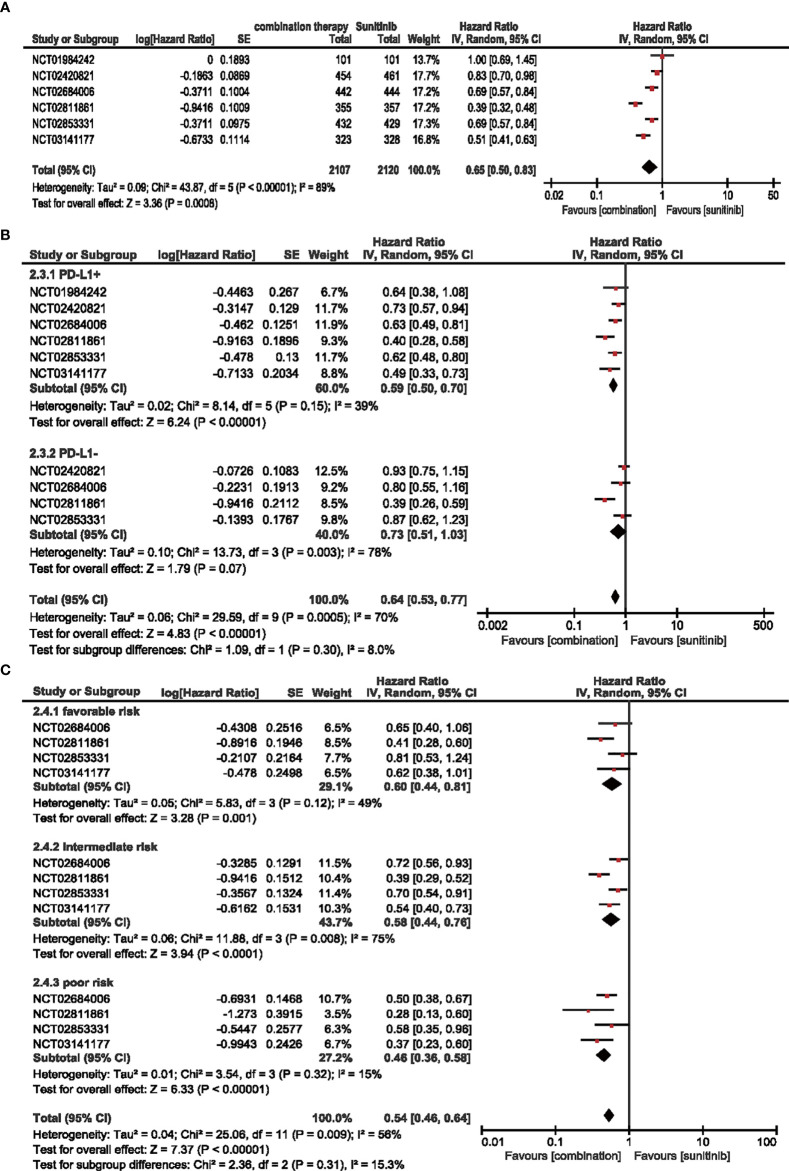
**(A)** Forest plot of PFS in patients treated with combination therapy of ICIs and anti-VEGF *vs.* sunitinib monotherapy. **(B)** Forest plot of PD-L1-positive and PD-L1-negative patients treated with combination therapy of ICIs and anti-VEGF *vs.* sunitinib monotherapy. **(C)** Forest plot of different IMDC-evaluated patients treated with combination therapy of ICIs and anti-VEGF *vs.* sunitinib monotherapy.

A total of 4,227 patients from six RCTs were included to analyze HR in the intention-to-treat population (ITT) and PD-L1-positive subgroups, and four RCTs were adopted for IMDC evaluation in age and sex subgroups. Compared with sunitinib monotherapy, the combination of ICIs and anti-VEGF therapy decreased the hazard ratio for PFS by 35% (HR = 0.65, 95% CI: 0.50–0.83, *p* = 0.0008; *I* ^2^ = 89%) **(** **Figure 3A** **)**.

##### 3.3.2.1 PFS in Patients With PD-L1-Positive Expression (≥1%)

In terms of the subgroups of PD-L1 expression, positive expression was associated with a steady 41% decrease in the hazard ratio (HR = 0.59, 95% CI: 0.50–0.70, *p* < 0.00001; *I* ^2^ = 39%), while negative expression did not show a statistically significant decrease (HR = 0.73, 95% CI: 0.51–1.03, *p* = 0.07; *I* ^2^ = 78%) **(** **Figure 3B** **)**.

##### 3.3.2.2 Subgroup Analysis PFS by Age, Sex, and IMDC

Similarly, no significant differences were detected in PFS for age and sex subgroups **(** **Supplementary Figure S4** **)**, and the hazard ratio decreased when the IMDC evaluation worsened. Moreover, combination therapy decreased the risk of progression by 40% (HR = 0.60, 95% CI: 0.44–0.81, *p* = 0.001; *I* ^2^ = 49%), 42% (HR = 0.58, 95% CI: 0.44–0.76, *p* < 0.0001; *I* ^2^ = 75%), and 54% (HR = 0.46, 95% CI: 0.36–0.58, *p* < 0.00001; *I* ^2^ = 15%) compared with sunitinib monotherapy in the favorable-, intermediate-, and poor-risk subgroups, respectively **(** **Figure 3C** **).**

#### 3.3.3 ORR

**Figure 4:**
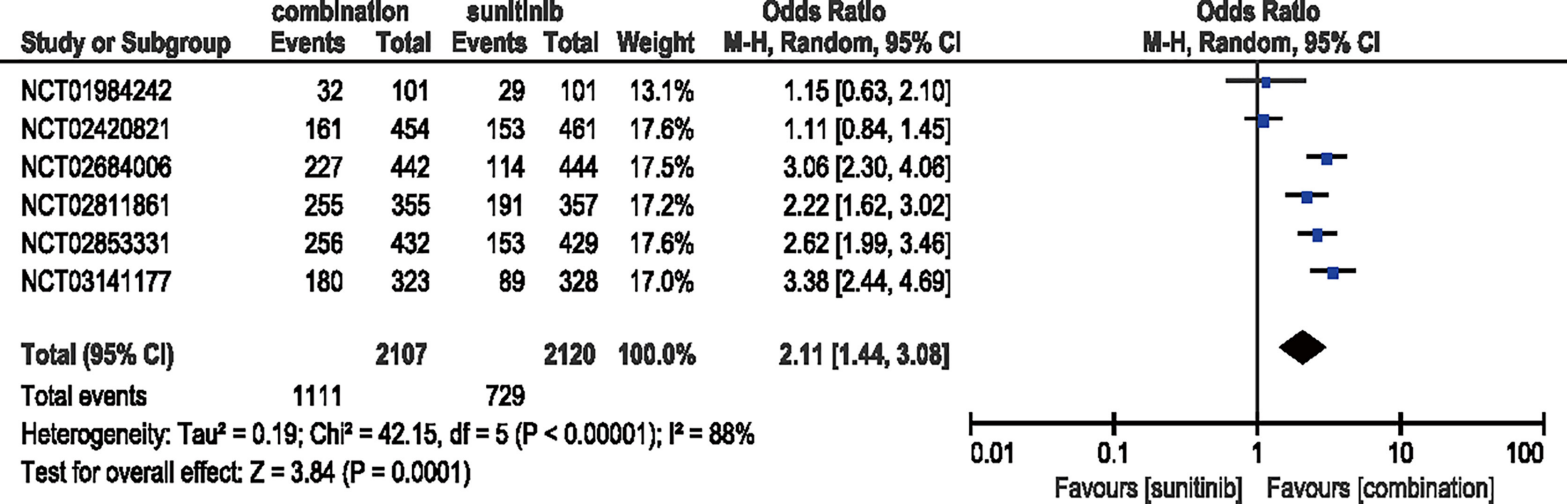
Forest plot of ORR in patients treated with combination therapy of ICIs and anti-VEFR *vs.* sunitinib monotherapy.

Six studies were included to analyze the ORR. Compared with sunitinib, combination therapy increased the ORR by 111% (ORR = 2.11, 95% CI: 1.44–3.08, *p* = 0.0001; *I* ^2^ = 88%) **(** **Figure 4** **)**.

### 3.4 Safety

Six randomized studies were adopted to calculate the RR of all- and high-grade (grades 3 to 5) AEs. According to previous research, some specific adverse events (e.g., proteinuria, arthralgia, rash, hypertension, diarrhea, stomatitis, HFSR, and dysgeusia) are monitored in RCC treatment and their presentation may lead to drug withdrawal. Therefore, we laid greater emphasis on these AEs and performed further meta-analysis to research the safety of combination therapy with ICIs and anti-VEGF versus sunitinib monotherapy. Except for proteinuria, all six RCTs were enrolled in the analysis of all-grade AEs and five RCTs were adopted in high-grade situations.

#### 3.4.1 Proteinuria

**Figure 5:**
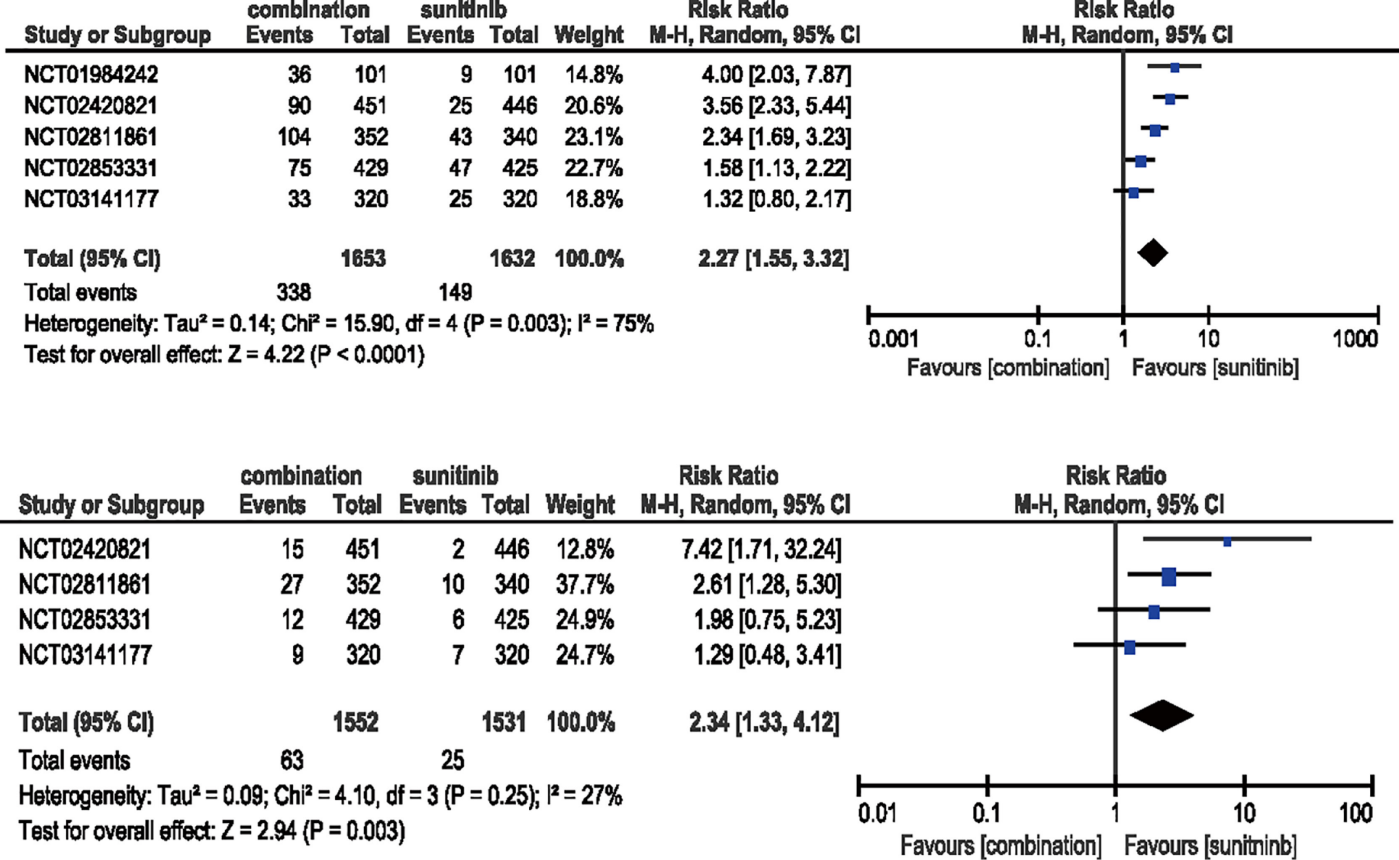
**(A)** Forest plot of all-grade proteinuria in patients treated with combination therapy of ICIs and anti-VEGF *vs.* sunitinib monotherapy. **(B)** Forest plot of high-grade proteinuria in patients treated with combination therapy of ICIs and anti-VEGF *vs.* sunitinib monotherapy.

Five studies were included in the analysis of all-grade, while four studies were included in high-grade proteinuria. Compared with sunitinib monotherapy, patients who received ICIs plus anti-VEGF therapy had significantly increased risk for all-grade proteinuria (RR = 2.27, 95% CI: 1.55–3.32, *p* < 0.0001; *I* ^2^ = 75%). The same trend was observed for high-grade proteinuria (RR = 2.34, 95% CI: 1.33–4.12, *p* = 0.003; *I* ^2^ = 27%) **(** **Figure 5** **)**.

#### 3.4.2 Arthralgia

**Figure 6:**
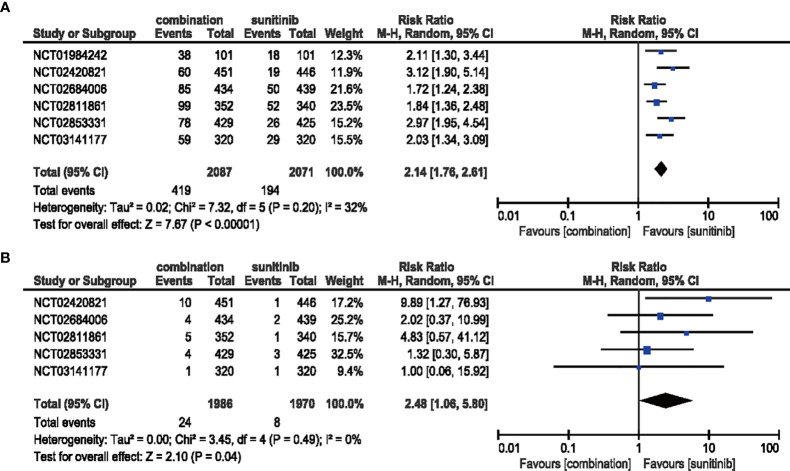
**(A)** Forest plot of all-grade arthralgia in patients treated with combination therapy of ICIs and anti-VEGF *vs.* sunitinib monotherapy. **(B)** Forest plot of high-grade arthralgia in patients treated with combination therapy of ICIs and anti-VEGF *vs.* sunitinib monotherapy.

The combination of ICIs and anti-VEGF therapy increased the risk of both all-grade (RR = 2.14, 95% CI: 1.76–2.61, *p* < 0.00001; *I* ^2^ = 32%) and high-grade arthralgia (RR = 2.48, 95% CI: 1.06–5.10, *p* = 0.04; *I* ^2^ = 0%) compared with sunitinib monotherapy **(** **Figure 6** **)**.

#### 3.4.3 Rash

**Figure 7:**
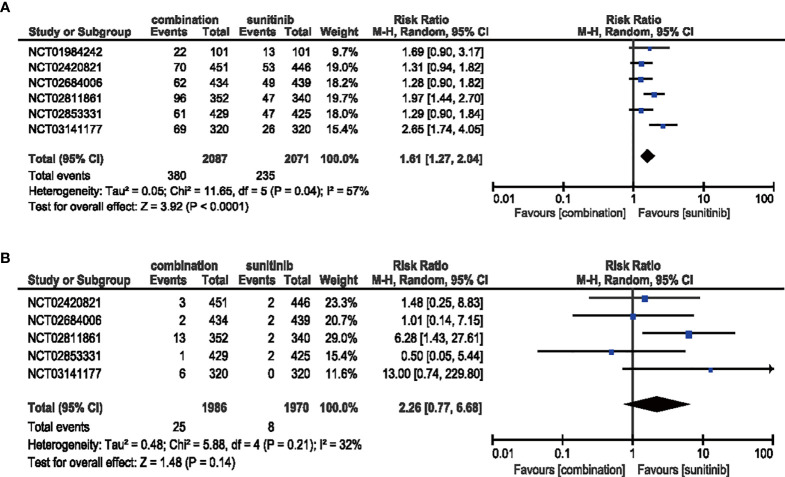
**(A)** Forest plot of all-grade rash in patients treated with combination therapy of ICIs and anti-VEGF *vs.* sunitinib monotherapy. **(B)** Forest plot of high-grade rash in patients treated with combination therapy of ICIs and anti-VEGF *vs.* sunitinib monotherapy.

All six studies demonstrated a significantly increased risk for all-grade rash when comparing combination therapy and sunitinib monotherapy (RR = 1.61, 95% CI: 1.27–2.04, *p* < 0.0001; *I* ^2^ = 57%), but this trend was not statistically significant for high-grade rash (RR = 2.26, 95% CI: 0.77–6.68, *p* = 0.14; *I* ^2^ = 32%) **(** **Figure 7** **).**

#### 3.4.4 Hypertension

**Figure 8:**
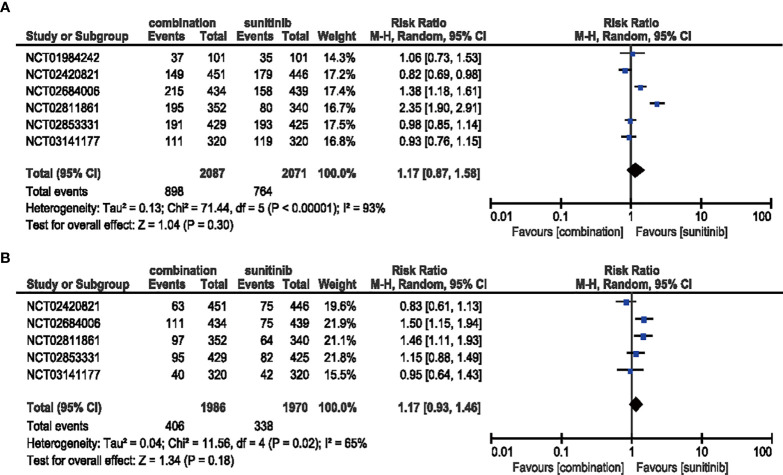
**(A)** Forest plot of all-grade hypertension in patients treated with combination therapy of ICIs and anti-VEGF *vs.* sunitinib monotherapy. **(B)** Forest plot of high-grade hypertension in patients treated with combination therapy of ICIs and anti-VEGF *vs.* sunitinib monotherapy.

The comparison between patients treated with combination therapy in all-grade (six studies included) (RR = 1.17, 95% CI: 0.87–1.58, *p* = 0.30; *I* ^2^ = 93%) and high-grade hypertension (five studies included) (RR = 1.17, 95% CI: 0.93–1.46, *p* = 0.18; *I* ^2^ = 65%) did not reveal any significantly increased risk, respectively **(** **Figure 8** **)**.

#### 3.4.5 Diarrhea

**Figure 9:**
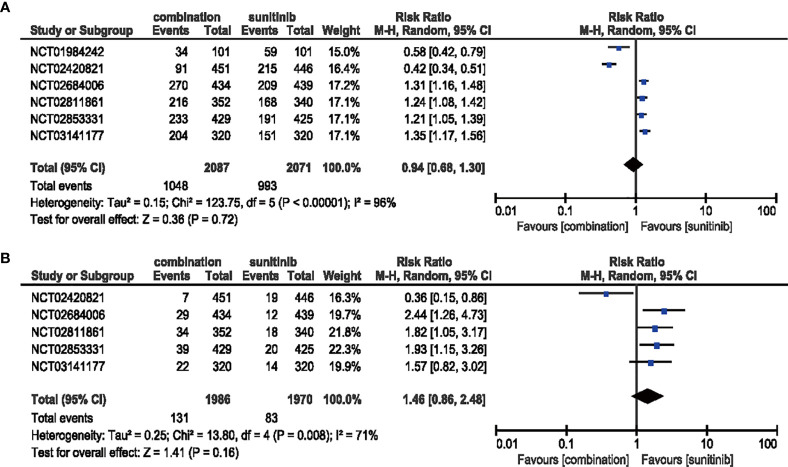
**(A)** Forest plot of all-grade diarrhea in patients treated with combination therapy of ICIs and anti-VEGF *vs.* sunitinib monotherapy. **(B)** Forest plot of high-grade diarrhea in patients treated with combination therapy of ICIs and anti-VEGF *vs.* sunitinib monotherapy.

Compared with sunitinib monotherapy, no evident difference was shown in the analysis of the all-grade (RR = 0.94, 95% CI: 0.68–1.30, *p* = 0.72; *I* ^2^ = 96%) or high-grade (RR = 1.46, 95% CI: 0.86–2.48, *p* = 0.16; *I* ^2^ = 71%) diarrhea **(** **Figure 9** **)**.

#### 3.4.6 Stomatitis

**Figure 10:**
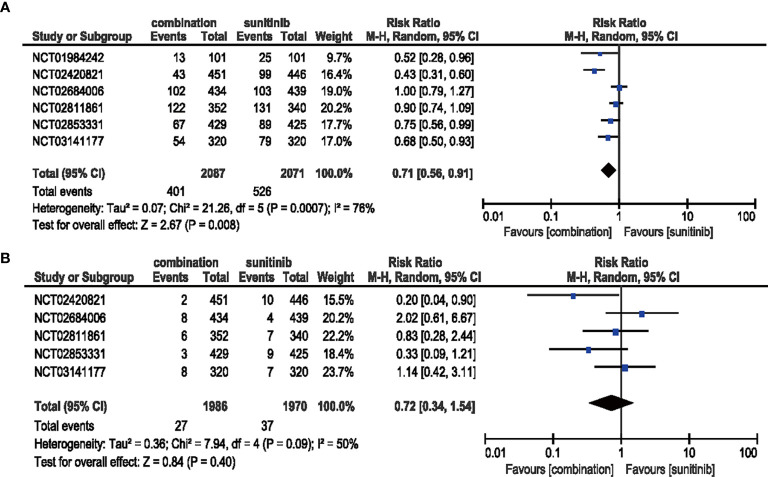
**(A)** Forest plot of all-grade stomatitis in patients treated with combination therapy of ICIs and anti-VEGF *vs.* sunitinib monotherapy. **(B)** Forest plot of high-grade stomatitis in patients treated with combination therapy of ICIs and anti-VEGF *vs.* sunitinib monotherapy.

Patients treated with combination therapy of ICIs and anti-VEGF showed a decreased risk (RR = 0.71, 95% CI: 0.56–0.91, *p* = 0.008; I^2^ = 76%) of all-grade stomatitis, while no significant benefit (RR = 0.72, 95% CI: 0.34-1.54, *p* = 0.40; *I* ^2^ = 50%) was obtained in high-grade stomatitis **(** **Figure 10** **)**.

#### 3.4.7 HFSR

**Figure 11:**
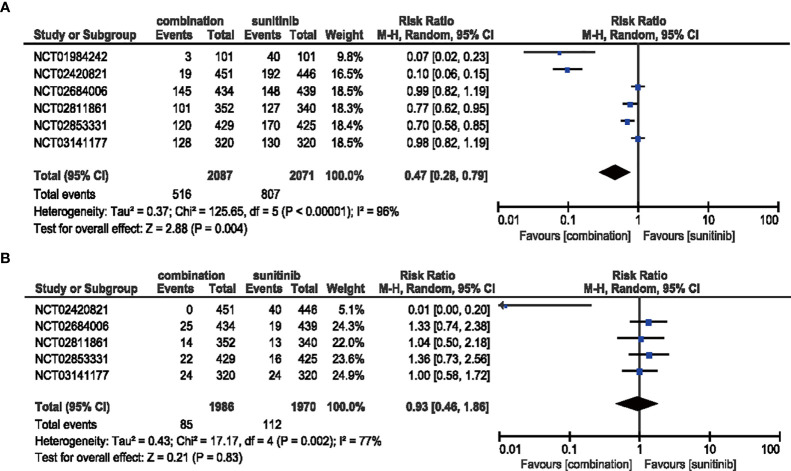
**(A)** Forest plot of all-grade HFSR in patients treated with combination therapy of ICIs and anti-VEGF *vs.* sunitinib monotherapy. **(B)** Forest plot of high-grade HFSR in patients treated with combination therapy of ICIs and anti-VEGF *vs.* sunitinib monotherapy.

The risk of all-grade HFSR decreased (RR = 0.47, 95% CI: 0.28–0.79, *p* = 0.004; *I* ^2^ = 96%) with the combination of ICIs and anti-VEGF therapy compared with sunitinib. However, no significant difference (RR = 0.93, 95% CI: 0.46–1.86, *p* = 0.83; *I* ^2^ = 77%) was detected in the same analysis of high-grade HFSR **(** **Figure 11** **)**.

#### 3.4.8 Dysgeusia

**Figure 12:**
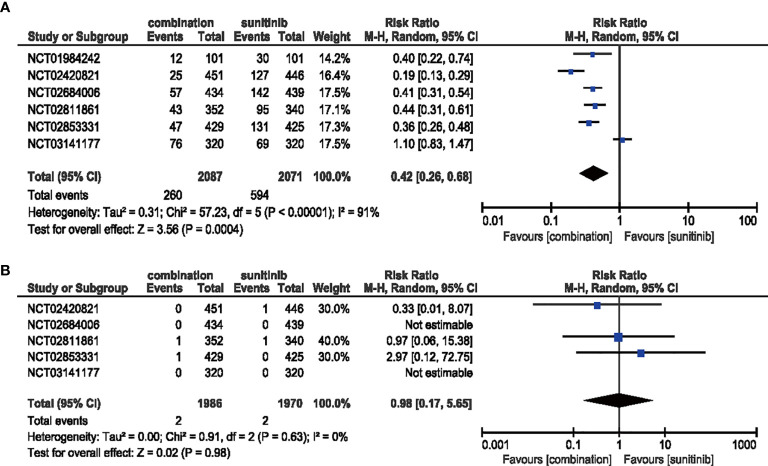
**(A)** Forest plot of all-grade dysgeusia in patients treated with combination therapy of ICIs and anti-VEGF *vs.* sunitinib monotherapy. **(B)** Forest plot of high-grade dysgeusia in patients treated with combination therapy of ICIs and anti-VEGFR *vs.* sunitinib monotherapy.

In the analysis of all-grade dysgeusia, the RR decreased (RR = 0.42, 95% CI: 0.26–0.68, *p* = 0.0004; *I* ^2^ = 91%) with the treatment of ICIs plus anti-VEGF therapy compared with sunitinib alone. However, the high-grade situation failed to support the same trend which may be due to inadequate incidence data (RR = 0.98, 95% CI: 0.17–5.65, *p* = 0.98; *I* ^2^ = 0%) **(** **Figure 12** **)**.

## 4 Discussion

Although considerable progress has been made in deducing the molecular mechanism of advanced RCC and relevant targeting drugs, the overall efficiency of these therapies is not yet satisfactory (33). Recently, the combination of targeting agents and immune checkpoint inhibitors to treat advanced RCC has been the top priority, owing to its potential additive or synergistic effects due to the high-level blockade of aberrant signaling (16, 34). Therefore, the current meta-analysis was performed to evaluate the therapeutic effect and associated AEs of combination therapy of ICIs and anti-VEGF versus sunitinib for first-line treatment of advanced RCC.

Combination therapy demonstrated tremendous efficacy compared with the traditional strategy of sunitinib monotherapy. According to our systematic analysis, the HRs were decreased in OS and PFS, and ORR was improved markedly. RCC is strongly linked to loss-of-function mutation in the *VHL* gene (35), which in turn, plays a vital role in reforming the tumor microenvironment (TME) with angiogenesis, pH regulation, and glucose transportation to suppress the chemotaxis and maturity of immune cells, thereby contributing to cancer survival. According to a previous study, the outstanding performance of the combination strategy can be attributed to normalizing the TME in the presence of cancer-derived VEGF and enhancing the function of cluster of differentiation eith positive T (CD8^+^T) cells to eliminate the cancer cells (36).

As mentioned before, the combination of ICIs and anti-VEGF therapy improves efficacy along with an increase in side effects. Hence, it is important to identify biomarkers to predict efficacy to further individualized precision therapy and balance the risk-benefit ratio. In the current study, the assumption that patients with PD-L1 expression could receive more benefits from ICI and anti-VEGF combination therapy was born out by the analysis of PFS and OS in the PD-L1-positive subgroup, while the HR of PFS in PD-L1-negative subgroup failed to reach statistical significance. Unfortunately, the HR of OS in the PD-L1-negative subgroup could not be calculated due to insufficient data. Other studies from Sun (37) and Buti (38) support the present conclusion that patients with PD-L1-positive expression might benefit more from combination therapy. However, we cannot assess whether PD-L1-negative expression is an obstructive factor to combination therapy-associated improvements in survival. Therefore, cautiousness is necessary regarding the use of PD-L1 expression level as a predictive factor for advanced outcomes (15). Indeed, PD-L1-negative patients might benefit from the combination therapy, and the heterogeneity of PD-L1 assessment criteria cannot be neglected (38). Moreover, PD-L1 expression levels would be needed to determine the specific threshold of the most effective combination therapy in the future. Furthermore, no obvious difference was detected in sex or age in the PFS subgroups. The slight discrepancy in the HR of OS was acceptable in the age subgroup because of a prolonged survival time for the younger population.

Another subgroup analysis according to IMDC evaluation was conducted. We found that the HR of the IMDC poor-risk population decreased (HR = 0.37; 95% CI: 0.26–0.54) compared with the intermediate-risk (HR = 0.65, 95% CI: 0.51–0.83) population in OS, while the favorable-risk population did not significantly benefit from combination therapy (RR = 0.90, 95% CI: 0.55–1.46). The latest NCCN guidelines recommend ICIs and anti-VEGF combination therapy including axitinib + pembrolizumab, cabozantinib + nivolumab, and lenvatinib + pembrolizumab as first-line therapy for RCC patients with relapse or stage IV disease, regardless of IMDC score (39). Similarly, the European Association of Urology (EAU) guidelines offer three combination regimens as mentioned above for treatment-naive patients with clear-cell metastatic RCC, without considering IMDC risk. In our study, PFS appears to benefit each IMDC subgroup but failed to convert to the prolonged OS. This might occur for several reasons. Firstly, the IMDC evaluation system is based on clinical features, while renal cell carcinoma is known for its high heterogeneity (40), which requires more precise molecular features to identify dominant tumor subtypes. Motzer et al. (41) identified seven molecular subsets associated with differential clinical outcomes to angiogenesis blockade alone or with a checkpoint inhibitor among 823 tumors from the IMmotion151 trial. In their study, tumors from favorable-risk patients were enriched in the angiogenic/stromal (No. 1) and angiogenic (No. 2) clusters, which exhibited higher expression of genes associated with the VEGF pathway. These findings provide a molecular explanation for the nonsignificant clinical outcomes to sunitinib monotherapy versus combined ICI + VEGF inhibition. Secondly, the baseline PD-L1 expression was lower in favorable-risk patients compared with the intermediate/poor-risk patients in the Checkmate 214 trial (42), in which IMDC favorable-risk patients failed to benefit from nivolumab plus ipilimumab combination therapy in contrast with sunitinib monotherapy. This might be an explanation for why the favorable-risk subgroup did not benefit more significantly from combination therapy compared with sunitinib in our study. Thirdly, extended follow-up from the Keynote 426 trial suggests that PFS in the favorable IMDC subgroup began to separate after 12 months and 70% of patients with favorable-risk disease in the pembrolizumab plus axitinib arm achieved an objective response compared with 50% of patients in the sunitinib group (43). Due to slow progress in favorable risk tumors, an overall survival benefit from the combination of immunotherapy and anti-VEGF therapy might require extended follow-up to present the “long tail” phenomenon of immunotherapy features. In summary, for IMDC favorable-risk patients, more molecular biomarkers besides PD-L1 are needed to select specific populations to guide clinical strategies better, and the existing data support combination therapy as more beneficial to IMDC intermediate- and poor-risk subgroups.

Proteinuria and hypertension are the most common AEs occurring in targeted therapy (15). Proteinuria is closely related to glomerular barrier dysfunction (44, 45). Since renal disorder is an independent risk factor for cardiovascular disease (46), combination therapy may increase the burden on the kidney, resulting in direct damage to renal tubules because podocytes and tubular cells widely express VEGF, leading to continuous drug accumulation and hypertension (15, 47). A significantly increased risk of developing hypertension was detected among RCC patients with continuous daily dosing compared with the intermittent dosing schedule (48, 49). Recently, anti-VEGF treatment was recognized as a potential trigger for an increased incidence of cardiovascular toxicity (50). Nonetheless, the management of hypertension remains controversial. Both enalapril and candesartan (angiotensin-converting enzyme inhibitor and angiotensin receptor blocker, respectively) were reported to inhibit myocardial angiogenesis induced by VEGF, while nifedipine (calcium channel blockers)-induced VEGF secretion (15, 51). Thus, the selection of medications may require a balance between side-effects and toxicity in conjunction with anti-VEGF.

Concerning arthralgia, the RR was increased by the combination therapy of ICIs and anti-VEGF therapy in both all-grade (RR = 2.14, 95% CI: 1.76–2.61) and high-grade AEs (RR = 2.48, 95% CI: 1.06–5.80). A previous study indicated that single nucleotide polymorphisms in the PD-1 gene were associated with susceptibility to rheumatoid arthritis, predisposing these patients to immune-mediated arthralgia (21). The clinical outcomes suggest that the rheumatoid factor should be verified and measures should be taken to prevent arthralgia.

Since the RR of any- and high-grade diarrhea did not reach statistical significance, it seems that the combination therapy did not increase the risk of developing diarrhea. However, the addition of ICIs increases the risk of diarrhea compared with chemotherapy alone (18). A further evaluation of toxicity is required as the underlying pathogenesis for sunitinib- or ICI-induced diarrhea is still unknown. Measures to cope with diverse AEs have been shown to exert a positive role in preventing the symptoms, including rehydration, electrolyte replacements, and loperamide (52). Interestingly, immunotherapy can be rechallenged after symptoms are resolved.

Rash and HFSR are the common AEs resulting from ICI monotherapy, and surprisingly, combination therapy had a reversal effect on them. The results of the current analysis suggested that the risk ratio of all-grade rash increased to 1.61 (95% CI: 1.27–2.04, *p* < 0.0001), while the combination therapy dramatically decreased the risk of all-grade HFSR (RR = 0.47, 95% CI: 0.28–0.79, *p* = 0.004). Reportedly, the application of TKI is strongly associated with all-grade HFSR. We also found that a specific combination therapy strategy (atezolizumab plus bevacizumab) held a tremendous potential to decrease the HFSR risk without TKI. Moreover, skin toxicity stands out among all types of AEs when patients are treated with ICIs (53). The blockade of PD-1 receptor by ICIs such as pembrolizumab, nivolumab, and avelumab triggers similar dermatological AEs (54). Further investigation is required to understand whether the decreased incidence of HFSR is attributed to the absence of TKI or different strategies of combination therapy. In order to improve QoL, a recent study highlighted adequate monitoring to maintain dose strength and prevent the worsening of lesions, including prescription of oral antihistamines and topical steroids with high potency (19).

Oral adverse events (OAEs) associated with TKIs and ICIs, are often overlooked (55). These events lead to significant consequences and disabilities, such as difficulty chewing and swallowing food (potentially leading to low QoL), dose modification, drug withdrawal induced by difficulty in administering oral medications, and a high risk of local and systemic infections. In the current study, the combination of ICIs and anti-VEGF decreased the risk of all-grade dysgeusia and stomatitis, contributing to continuous drug application. Dysgeusia and stomatitis were improved rapidly at untreated intervals and systematic management. However, these might recur with additional doses of the target agent. Since the discontinuation of treatment-induced OAEs has been studied sparsely, the development of systematic management can ensure safety outcomes (18).

Taking the current analysis into consideration, we have identified a series of problems that remain to be solved. Firstly, it is still unclear how ICIs and anti-VEGF can be best applied and combined in systematic therapy. Notably, the specific combination of atezolizumab and bevacizumab described in the NCT01984242 and NCT02420821 trials showed outstanding performance in managing all-grade AEs (stomatitis, diarrhea, and HFSR), indicating that specific medication combination may exert a positive impact on the safety. However, due to the limited quantity and quality of the included studies, we could not perform subgroup analysis on the influence of a specific combination of ICIs or anti-VEGF medication. Secondly, it is unclear whether the survival benefits outweigh the potentially increased risk for AEs with concurrent or sequential therapy in RCC patients. Thirdly, in combination therapy, discontinuation is usually caused by high-grade or severe AEs. Standardized solutions should be studied and adopted to minimize the negative impact of some common AEs. A series of dermatological suggestions in a previous study (19) indicated the potential to overcome AEs and sustain continuous administration. Finally, since all patients may benefit from combination therapy, precise biomarkers are required to optimize the clinical efficacy between combination and monotherapy.

## 5 Strengths and Limitations

The strengths of this meta-analysis are as follows: to the best of our knowledge, this is the most comprehensive study to evaluate the efficacy and safety of ICIs combined with anti-VEGF therapy. Moreover, all identified studies were RCTs with high quality and low-to-moderate risk of bias. The current meta-analysis delved into the differences between PD-L1-positive/negative and ITT subsets to determine the optimal population for progressive or metastatic RCC based on PD-L1 expression. The optimal clinical decisions were based on the common, specific, and representative AEs; the differences in severity were also explored.

Nonetheless, as only six RCTs were included in the current meta-analysis, data were insufficient for specific subgroup analysis. Therefore, excluding the influence of drug classification and identifying optimal patients benefiting from combination agents requires further study, and the observed heterogeneity cannot be explained. The random-effects model might minimize some of these issues and balance the weight of various sample sizes in the trials. Additionally, PD-L1 expression scores were divided into positive and negative expression to investigate the optimal benefit of combination therapy. However, it was not sufficient for a primary conclusion due to the absence of cutoff values in PD-L1 expression.

## 6 Conclusion

The current analysis showed that ICIs combined with anti-VEGF improved the prognosis in patients with RCC. However, for OS, existing evidence failed to prove a better prognosis for favorable-risk patients evaluated by IMDC. However, the incidence of specific AEs increased obviously compared with monotherapy. The contradictory performance for different AEs is a serious issue that may prevent standardized clinical administration. Thus, we cautiously conclude that combination therapy can be widely utilized in the future with the development of optimal administration and systemic AE management. Additionally, individualized therapy should be intensively studied to achieve the best benefit-risk ratio in clinical application.

## Data Availability Statement

The datasets presented in this study can be found in online repositories. The names of the repository/repositories and accession number(s) can be found in the article/**Supplementary Material**.

## Author Contributions

JY, LT, and HZ contributed to conception and design of the study. LT organized the database, performed the statistical analysis, and wrote the first draft of the manuscript. LT, JY, and HZ wrote sections of the manuscript. All authors contributed to manuscript revision, read, and approved the submitted version.

## Funding

This work was supported by a grant from the College Students Science Innovation Project of Capital Medical University (XSKY2021247).

## Conflict of Interest

The authors declare that the research was conducted in the absence of any commercial or financial relationships that could be construed as a potential conflict of interest.

## Publisher’s Note

All claims expressed in this article are solely those of the authors and do not necessarily represent those of their affiliated organizations, or those of the publisher, the editors and the reviewers. Any product that may be evaluated in this article, or claim that may be made by its manufacturer, is not guaranteed or endorsed by the publisher.
