# Supplementary material for: Balancing the Risk-Benefit Ratio of Immune Checkpoint Inhibitor and Anti-VEGF Combination Therapy in Renal Cell Carcinoma: A Systematic Review and Meta-Analysis
Source: Front Oncol. 2021 Oct 14;11:739263. doi: 10.3389/fonc.2021.739263 (PMC8552014; doi:10.3389/fonc.2021.739263)
Supplement: Supplementary file 1 [file DataSheet_1.docx]

***Supplementary material***

**Supplementary Table 1.** The PRISMA Statement for Meta-analysis

| Section/topic | Item No | Checklist item | Page  No |
| --- | --- | --- | --- |
| Title |  |  |  |
| Title | 1 | Balancing the risk-benefit ratio of immune checkpoint inhibitor and anti-VEGF combination therapy in renal cell carcinoma: a systematic review and meta-analysis | 1 |
| Abstract |  |  |  |
| Structured summary | 2 | Shown on the text part. | 1-2 |
| Introduction |  |  |  |
| Rationale | 3 | Both the combination therapy of ICIs and anti-VEGFR and Sunitinib monotherapy have been recommended in the revised National Comprehensive Cancer Network (NCCN) guidelines. | 2-3 |
| Objectives | 4 | P: patients with renal cell carcinoma  I: combination therapy of ICIs (immune checkpoint inhibitors) and anti-VEGF (vascular endothelial growth factor) medication  C: Sunitinib monotherapy  O: overall survival (OS), progression-free survival (PFS), objective response ratio (ORR)  S: meta-analysis | 3-4 |
| Methods |  |  |  |
| Protocol and registration | 5 | DOI：10.37766/inplasy2021.3.0104 | —— |
| Eligibility criteria | 6 | 1) Patients who were diagnosed with RCC or had previously untreated advanced RCC with a clear-cell component and at least one measurable lesion according to Response Evaluation Criteria in Solid Tumors (RECIST); 2) Karnofsky performance status score of at least 70 (scores range from 0 to 100, with lower scores indicating greater disability); 3) Adults(18years old or older); 4) Adequately controlled blood pressure, with or without medications; and adequate organ function; 5) Patients without previous systemic therapy for advanced disease; 6) Studies reported with efficacy, including overall survival (OS), progression-free survival (PFS) and objective response rate (ORR) and associated AEs; 7) Randomized controlled trial studies; 8) when results from an RCT was reported and analyzed more than once, the primary data will be included. | 5 |
| Information sources | 7 | Pubmed; Embase; Cochrane | 4-5 |
| Search | 8 | The search terms were as follows: "renal carcinoma/exp" and "randomized controlled trial/exp" and ("vasculartropin/exp" or "anti-angiogenesis/exp" or "angiogenesis inhibitor/exp") and ("immune checkpoint inhibitor/exp" or "programmed cell death protein 1/exp" or "programmed cell death ligand protein 1/exp" or "cytotoxic T-lymphocyte-associated protein 4/exp") and "human/exp". No language limitation was applied, and all adopted studies were screened manually on the reference list and other relevant articles. | 4-5 |
| Study selection | 9 | Shown on the Figure 1 | 7 |
| Data collection process | 10 | Collect accessible data and form a table | 7-8 |
| Data items | 11 | PICOS; Year; Author; Treatment arm; Number of patients; Baseline data; | 5-6 |
| Risk of bias in individual studies | 12 | Selection bias; Selection bias; Performance bias; Detection bias; Attrition bias; Reporting bias; | 10 |
| Summary measures | 13 | Hazzard ratio (HR) of Overall survival (OS), Progression-free survival (PFS) and Objective response rate (ORR)，Risk ratio of adverse events. | 6 |
| Synthesis of results | 14 | Subgroup analysis; Random effect model. | 6 |
| Risk of bias across studies | 15 | Bias analysis in Figure 1 | 6 |
| Additional analyses | 16 | Subgroup analysis of age, sex, IMDC assessment for OS and PFS | 6 |
| Results |  |  |  |
| Study selection | 17 | A total of 3042 studies were identified, of which 1006 were duplicates. We scanned titles and abstracts and excluded 1897 articles for not meeting the inclusion criteria. Having obtained full-text articles for 139 citations, we excluded 133 for non-RCT. At last, six articles involving 4227 participants were adopted in this systematic review and meta-analysis. The selection flow diagram is shown in Figure 1A. | 6-7 |
| Study characteristics | 18 | Table 1 | 8 |
| Risk of bias within studies | 19 | The Inverse-Variance (I-V) pooling model was applied to analyze OS, PFS and ORR, while the Mantel-Haenszel (M-H) pooling model was adopted in the analysis of adverse events. | 6 |
| Results of individual studies | 20 | Sequence number 3.3-3.4 | 10-14 |
| Synthesis of results | 21 | Sequence number 3.3-3.4 | 10-14 |
| Risk of bias across studies | 22 | The risk of bias was assessed by the Cochrane Collaboration and was classified as "low," "unclear," or "high" in several areas. All six trials have an unclear risk of performance bias because of the design of open label. With the absence of allocation design and independent assessment institution results in the trial (NCT01984242), the selection and detection bias are assessed unclear risk. | 6 |
| Additional analysis | 23 | The subgroup analysis of OS and PFS are shown in the sequence number of 3.3.1.1-3.3.1.2 and 3.3.2.1-3.3.2.2 | 10-12 |
| Discussion |  |  |  |
| Summary of evidence | 24 | Comprehensive discussion was performed in this article. | 14-19 |
| Limitations | 25 | As only six RCTs were included in the current meta-analysis, data was insufficient for specific subgroup analysis. Therefore, excluding the influence of drug classification and identifying optimal patients benefiting from combination agents require a follow-up, and the heterogeneity could not be explained. The random-effects model might minimize some of these issues and balance the weight of various sample sizes trials. Additionally, the PD-L1 expression scores were divided into positive and negative expressions to investigate the optimal benefit of combination therapy. However, it was not optimal for a primary conclusion due to the absence of many cutoff values in PD-L1 expression. | 19-20 |
| Conclusions | 26 | The current analysis showed that ICIs combined with anti-VEGFR promoted the prognosis in patients with RCC. But for OS, existing evidence failed to prove a better prognosis for ‘favorable’ patients evaluated by IMDC. However, the incidence of specific AEs increased obviously compared with monotherapy. The contradictory performance in different AEs had high costs and is not an accurate unification in standardized clinical administration. Thus, we cautiously concluded that the combination therapy could be widely utilized in the future with the development of optimal administration and systemic AEs management. Also, individualized therapy should be under intensive focus to achieve the best benefit-risk ratio in clinical application. | 20 |
| Funding |  |  |  |
| Funding | 27 | This work was supported by a grant from College Students Science Innovation Project of Capital Medical University (XSKY2021247). | 21 |


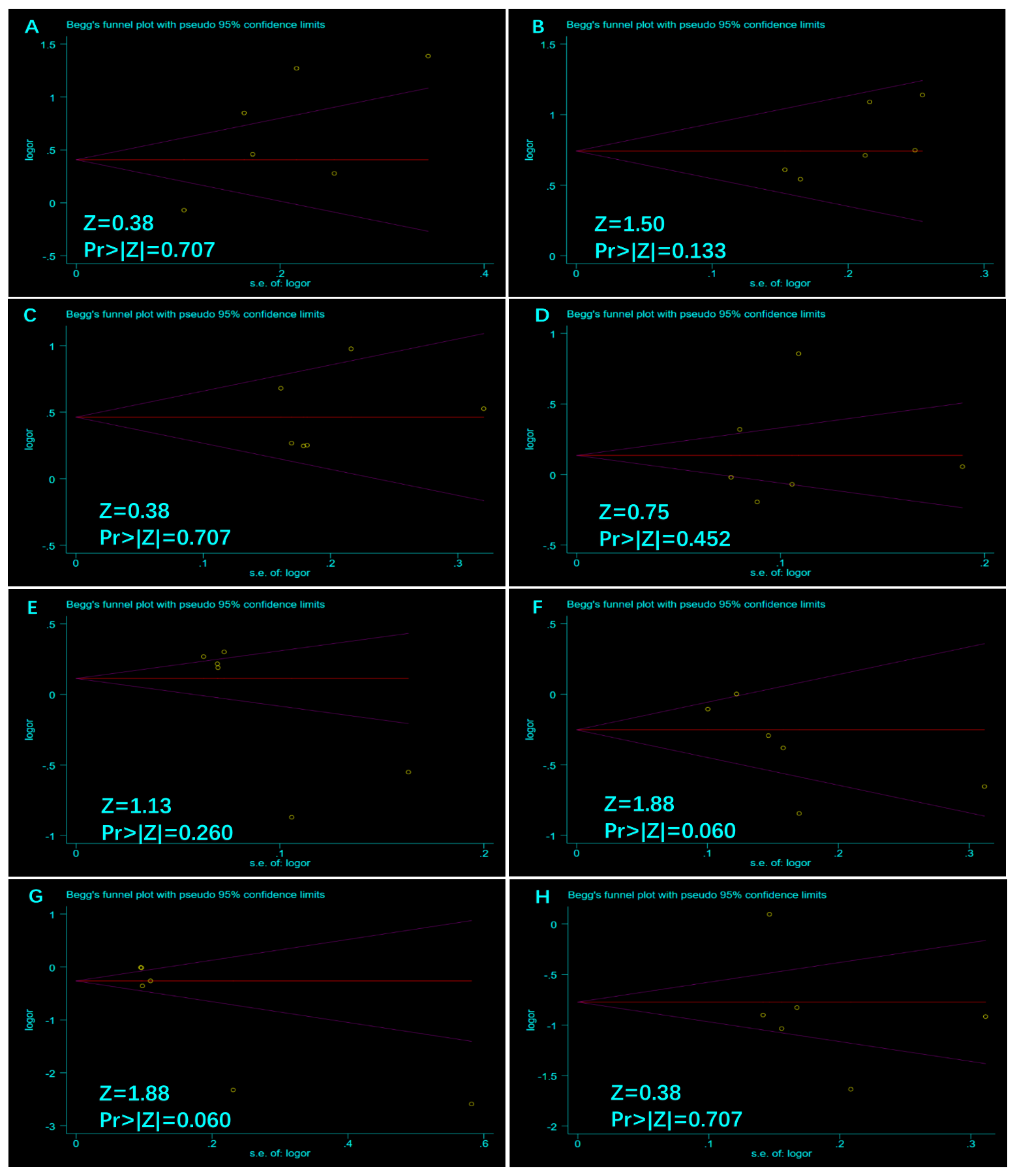


**Supplementary Figure 1.** Begg’s publication bias plots and their P values of all adverse events. (A)Proteinuria. (B)Arthralgia. (C)Rash. (D)Hypertension. (E)Diarrhea. (F)Stomatitis. (G)HFSR. (H)Dysgeusia.


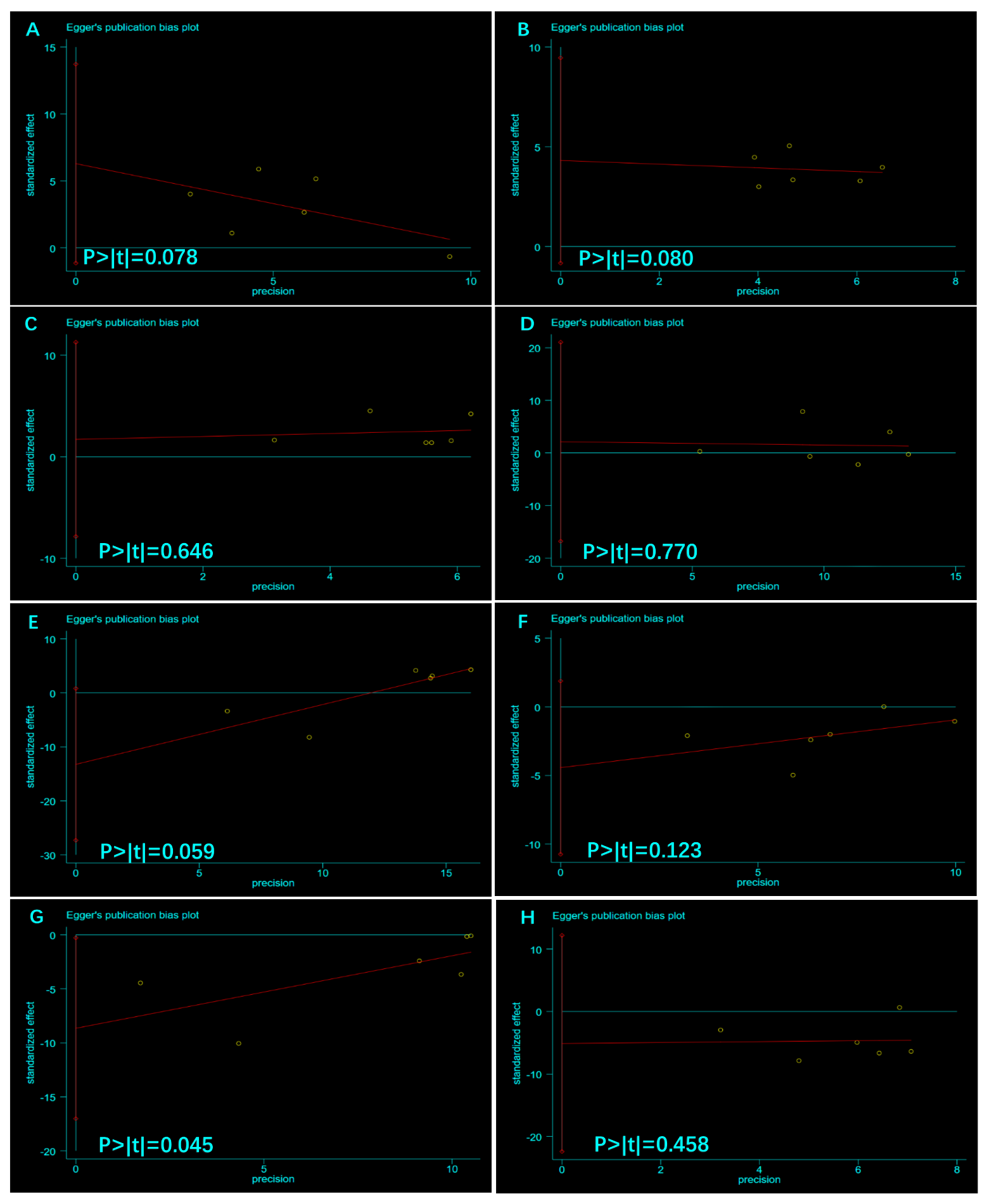


**Supplementary Figure 2.** Egger’s publication bias plots and their P values for all adverse events.

(A)Proteinuria. (B)Arthralgia. (C)Rash. (D)Hypertension. (E)Diarrhea. (F)Stomatitis. (G)HFSR. (H)Dysgeusia.


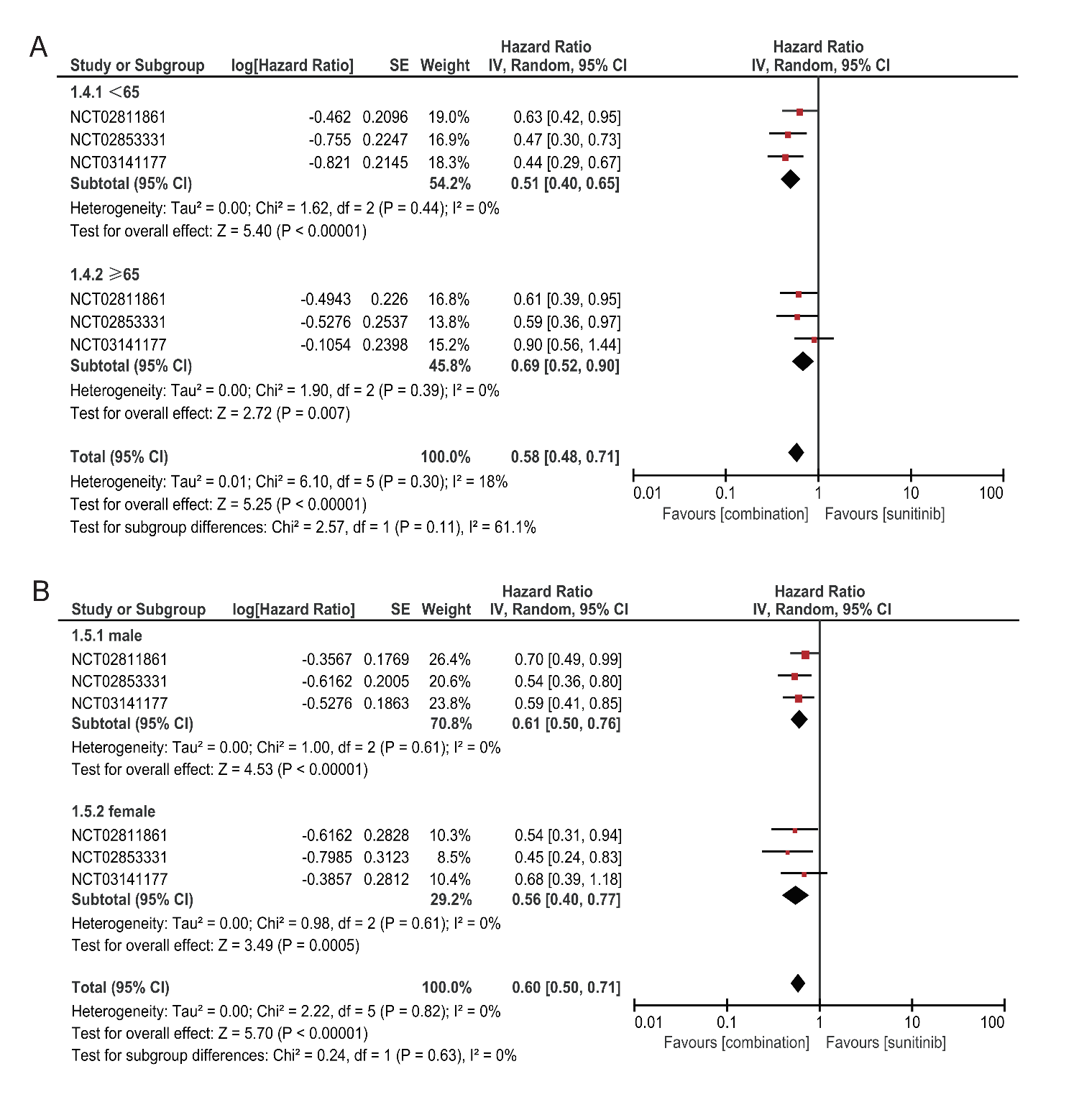


**Supplementary Figure 3.** (A) Forest plot of OS in different age patients treated with combination therapy of ICI and anti-VEGF vs. sunitinib monotherapy. (B) Forest plot of OS in different sex patients treated with combination therapy of ICI and anti-VEGF vs. Sunitinib monotherapy.


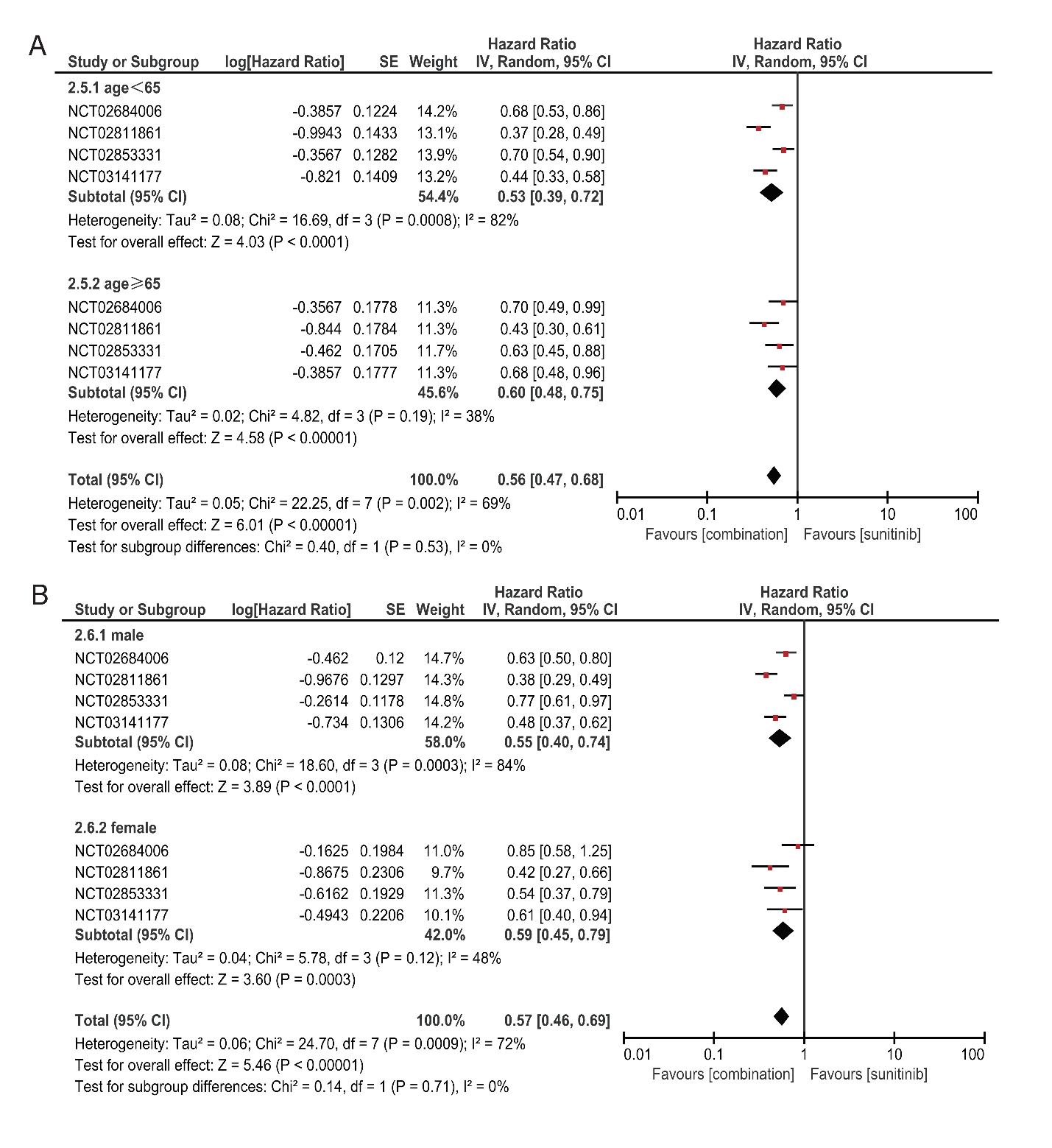


**Supplementary Figure 4.** (A) Forest plot of PFS in different age patients treated with combination therapy of ICI and anti-VEGF vs. Sunitinib monotherapy. (B) Forest plot of PFS in different sex patients treated with combination therapy of ICI and anti-VEGF vs. Sunitinib monotherapy.
